# Study of the Plasticization Effect of 1-Ethyl-3-methylimidazolium Acetate in TPS/PVA Biodegradable Blends Produced by Melt-Mixing

**DOI:** 10.3390/polym15071788

**Published:** 2023-04-04

**Authors:** Jennifer M. Castro, Mercedes G. Montalbán, Daniel Domene-López, Ignacio Martín-Gullón, Juan C. García-Quesada

**Affiliations:** 1 Chemical Engineering Department, University of Alicante, Apartado 99, 03080 Alicante, Spain; 2 Institute of Chemical Process Engineering, University of Alicante, Apartado 99, 03080 Alicante, Spain; 3 Chemical Engineering Department, Faculty of Chemistry, Regional Campus of International Excellence “Campus Mare Nostrum”, University of Murcia, 30071 Murcia, Spain

**Keywords:** thermoplastic starch/polyvinyl alcohol blend, ionic liquid, 1-ethyl-3-methylimidazolium acetate, melt-mixing, plasticizer

## Abstract

The first step towards the production and marketing of bioplastics based on renewable and sustainable materials is to know their behavior at a semi-industrial scale. For this reason, in this work, the properties of thermoplastic starch (TPS)/polyvinyl alcohol (PVA) films plasticized by a green solvent, as the 1-ethyl-3-methylimidazolium acetate ([Emim^+^][Ac^−^]) ionic liquid, produced by melt-mixing were studied. These blends were prepared with a different content of [Emim^+^][Ac^−^] (27.5–42.5 %wt.) as a unique plasticizer. According to the results, this ionic liquid is an excellent plasticizer due to the transformation of the crystalline structure of the starch to an amorphous state, the increase in flexibility, and the drop in T_g_, as the [Emim^+^][Ac^−^] amount increases. These findings show that the properties of these biomaterials could be modified in the function of [Emim^+^][Ac^−^] content in the formulations of TPS, depending on their final use, thus becoming a functional alternative to conventional polymers.

## 1. Introduction

Ionic liquids (ILs) are gaining interest in the field of plastic production because they have the potential to act as plasticizers in polymer matrices [1] due to properties such as high thermal and chemical stability, non-flammability, low vapor pressure, tunable solubility, and good hydraulic properties [2,3,4,5,6,7]. In addition, they can be found as liquids in a wide range of temperatures because they are organic salts with melting points below 100 °C, formed by a bulky inorganic cation (with a low degree of symmetry) and as an anion resulting in a little compact structure with large and non-uniform ions [8,9]. Some authors have previously reported the use of ILs as plasticizers of different conventional polymers. Scott et al. [10] studied the properties of poly(methyl methacrylate) (PMMA) plasticized by the IL 1-butyl-3-methylimidazolium hexafluorophosphate and concluded that the material was comparable with those that contain conventional plasticizers, noting that the low volatile IL created a more flexible polymer over a long lifetime which prevented the material from brittleness. Similar conclusions were obtained by Rahman et al. [9], who reported that the addition of a variety of ILs to different PVC formulations improved mechanical properties (increasing the flexibility) at the same time that films had a longer life with a decrease of plasticizer migration.

In industry, ILs are commonly used as catalysts for the production and storage [11] of energy, or as an extracting agent for organic compounds [12]. ILs can be considered “green” solvents because they are environmentally friendly, with low explosion risk, and with easily recovered and recycled procedures [13,14]. Therefore, ILs can play an interesting role in the new environmental context, where research oriented toward the development of new materials from renewable and sustainable sources is experiencing significant growth. In this sense, the plastic industry is one of the most affected sectors and exchanging conventional fossil-based polymers with renewable polymers is a solution for some up-to-date problems due to the enormous consumption of “limited” resources, the problems related to plastic waste mismanagement [15,16,17,18], and their harmful effects on the environment [19,20,21].

In the past, some researchers have investigated the effect of ILs as a solvent in the research of biodegradable and renewable materials. However, recently, ILs have been used to study their plasticizer effect in biodegradable materials [22,23,24,25,26,27,28,29,30,31], with starch being one of the most promising materials due to its abundance, low cost, and biodegradability [15]. Starch is a biopolymer composed of two polysaccharides: amylose and amylopectin [32,33]; and the presence of plasticizers is needed in order for it to be transformed into thermoplastic starch (TPS) [34] by a gelatinization process [35]. There are a number of studies on the use of ILs as plasticizers in biodegradable starch blends. Wilpiszewska et al. [8] concluded that the presence of ILs on TPS could decrease the glass transition temperature more than when using glycerol, suggesting the better plasticization efficiency of ILs in comparison to polyols which are conventional plasticizers for these biomaterials [36,37,38,39]. These conclusions were supported by Xie et al. [40], Domene-López et al. [41], and Abera et al. [42], who studied the behavior of films prepared with maize, potato, and anchote starch, respectively, using 1-ethyl-3-methylimidazolium acetate as a plasticizer by a solution casting method in the presence of water.

The use of 1-ethyl-3-methylimidazolium acetate ([Emim^+^][Ac^−^]), among other ILs, as a TPS plasticizer is interesting because it has a more biodegradable character and is less toxic than other ILs due to its carboxylic anion [43,44]. Furthermore, it is capable of forming chemical bonds with CO_2_ [45]. Chen et al. [46] studied the process of atmospheric water sorption and CO_2_ capture in a system of [Emim^+^][Ac^−^] and biopolymers (cellulose, chitin, and chitosan). Their findings indicate that these biomaterials could be employed for food packaging and storage applications. In our research group, we have studied the prospective use of [Emim^+^][Ac^−^] with different botanical sources of starch, and have concluded that the starch characteristics are crucial in order to obtain good blend properties and the potato blend values were especially very promising [47]. Moreover, the incorporation of multi-walled carbon nanotubes (MWCNT) to the starch blends plasticized by [Emim^+^][Ac^−^] showed great electrical conductivity, proving that these types of biomaterials could be used in a wide range of applications, such as lithium batteries, fuel cells, or dye-sensitized solar cells [26,41].

The use of the casting method is common in laboratory-scale TPS research because this allows for the study of the starch films plasticized under ideal gelatinization conditions, and promoted by an excess of plasticizer. Nevertheless, for the purposes of industrial research, these materials should be processed in semi-industrial scale equipment, considering real conditions, and without an excess of plasticizer. For example, Sankri et al. [25] prepared TPS from maize starch plasticized by water, glycerol, and 1-butyl-3-methylimidazolium chloride using melt processing and they confirmed that the IL had strong plasticized effects through the mechanical property values achieved. Decaen et al. [48] studied the rheology properties of TPS from maize starch plasticized by three ILs in the presence of water by melt rheology at 120 °C. They concluded that the [Emim^+^][Ac^−^] is a promising plasticizer due to the lowest shear viscosity and limited macromolecular degradation. 

TPS is usually formulated with water since it is considered the best starch plasticizer, but its use has several limitations such as the evaporation of the water during the process (temperatures above 100 °C are needed in the industrial process), and the interaction between the water and the ILs acetate anions can limit the plasticization effect of the IL studied [49,50]. TPS pure films are brittle due to the strong chain interactions of the starch polymer via hydrogen bonding which increases the cohesion forces of the matrix [51]. Therefore, the use of other adjuvant polymers such as polyvinyl alcohol (PVA) [52] is a good option to confer resistance to starch-based materials. PVA is currently employed in the food, medical, and hygiene sectors, among others [15]. PVA is a synthetic polymer with a high degree of biocompatibility, solubility in water, chemical resistance, biodegradability, and other notable physical properties by its hydroxyl groups, which promotes the formation of hydrogen bonds [53] and, in addition, this thermoplastic has remarkably superior features as an oxygen barrier, compared with the other biopolymers [54]. 

Thus, the aim of this work is the study of the plasticization effect of [Emim^+^][Ac^−^] in thermoplastic starch/PVA blends by melt-mixing using semi-industrial scale equipment. Samples have been prepared without water to prevent interaction with the ILs. Mechanical and hydration properties, migration degree of plasticizer, dynamic mechanical analysis, morphology, and XRD and ATR-FTIR spectra of the TPS films are discussed to determine the potential capability of these materials to replace synthetic and non-biodegradable polymers. To the best of our knowledge, no studies can be found in the literature about starch/PVA mixtures plasticized by a single plasticizer consisting in IL, without an additional water supply, and produced by melt-mixing.

## 2. Materials and Methods

### 2.1. Materials

The potato starch was provided by Across Organics (Geel, Belgium) and characterized in our previous works [47,55]. PVA hydrolyzed (Mw: 89,000–98,000 Da) was purchased from Sigma-Aldrich (Madrid, Spain). The ionic liquid 1-ethyl-3-methylimidazolium acetate ([Emim^+^][Ac^−^]) (>95% purity) was supplied by loLiTec-Ionic Liquids Technologies GmbH (Heilbronn, Germany). Zinc stearate, used as a lubricant, was also provided by Sigma-Aldrich (Madrid, Spain). All chemicals were used without further purification. 

### 2.2. Preparation of the Starch/PVA Films Using the Ionic Liquid as Plasticized

Starch films were prepared by the method of starch melt-compounding previously described by Domene-Lopéz et al. [15], with some modifications. The processing was carried out with the HAAKE TM PolyLab TM QC Modular Torque Rheometer (ThermoFisher Scientific, Waltham, MA, USA). The samples were processed at 130 °C for 14 min; the first 8 min at 50 rpm at the rest of the time at 100 rpm. The blend obtained was then hot pressed at 160 °C for 15 min at a pressure of 10 tons obtaining a 1 mm thick sheet. Samples were stored in a controlled atmosphere of relative humidity of 50% for 48 h prior to their further characterization, making sure to avoid the TPS properties being changed by the humidity effect [56].

Starch, PVA, and ionic liquid were manually premixed at room temperature for 3 min and then introduced in the HAAKE rheometer. The weight ratio between the potato starch and PVA in the sample was 1:1, adding different amounts of ionic liquid as plasticizer: 27.5, 30, 32.5, 37.5, 40, and 42.5 %wt. of ionic liquid; the studied formulations were labeled as IL_27.5, IL_30, IL_32.5, IL_37.5, IL_40, and IL_42.5, respectively, and 0.5 %wt. of zinc stearate was also added to all blends.

### 2.3. Characterization of the Films

#### 2.3.1. Scanning Electron Microscopy (SEM) Analysis

SEM images were obtained with a Hitachi Scanning Electron Microscope (Hitachi S3000N, Tokyo, Japan) using an accelerating voltage of 10 kV. To observe the microstructure and morphology, the samples were cryo-fractured by immersion in liquid nitrogen and cooled with gold.

#### 2.3.2. Mechanical Properties

Mechanical properties of the films were determined with an Instron 3344 Universal Test Instrument (Norwood, MA, USA) equipped with a 2000 N load cell and operated at 25 mm/min following ASTM D882-12(2012) [57] standard recommendations. Each film was cut into dumbbell-shaped specimens. The tensile properties studied were Young’s Modulus (MPa), tensile strength at break (MPa), and elongation at break (%), which were calculated using the average thickness of the specimen and at least eight specimens were tested.

#### 2.3.3. Hydration Properties

The hydration properties were determined following the method previously described in the literature [15,41,47,55,58]. The films were cut into 1 × 1 cm² specimens. On the one hand, the water content was determined by measuring the lost weight after drying the samples in an oven at 110 °C for 5 h. The moisture content was calculated using Equation (1) and was expressed as a percentage (grams of water in 100 g of sample):

(1) $$H\left(\%\right)=\left(\frac{m_{0}-m_{1}}{m_{0}}\right)\times 100$$

where *m*_0_ is the initial mass and *m*_1_ is the mass after drying.

On the other hand, the solubility in the samples of water was determined by placing the films individually in 10 mL tubes filled with 9 mL of distilled water, which were capped and stored at 25 °C for 24 h. After that, the samples were taken out and dried again at 110 °C for 5 h. Solubility in water was calculated using Equation (2):

(2) $$Solubility\;\left(\%\right)=\left(\frac{m_{0}-m_{f}}{m_{0}}\right)\times 100$$

where *m_f_* is the final dry mass.

The water content and solubility values were taken as the average of at least five repetitions.

#### 2.3.4. Migration Degree

The degree of migration of plasticizer to the surface of films is determined as the weight loss of the sample after the migration test, which was carried out following a modified procedure based on methods described by Marcilla et al. [59] and Rahman et al. [9]. The films were cut into small circles with dimensions of 7 mm in diameter and 1 mm thickness, which were placed between two Petri dishes with absorbent papers and kept under a pressure of 16.5 kPa in an oven at 60 °C for 1 week. The values of plasticizer migration were obtained by taking out the samples at different times (1, 2, 5, and 7 days), and then calculating the weight loss using Equation (3):

(3) $$Migration\;Degree\;\left(\%\right)=\left(\frac{m_{0}-m_{f}}{m_{0}}\right)\times 100$$

where *m*_0_ and *m_f_* are the initial and final weights of the sample.

The results of the migration of plasticized were obtained from an average of at least three repetitions.

#### 2.3.5. Attenuated Total Reflection-Fourier Transform Infrared (ATR-FTIR) Spectroscopy

A Bruker Spectrometer (IFS 66/S model, Ettlingen, Germany) with an ATR accessory was used to obtain the ATR-FTIR spectra of films between 400 and 4000 cm^−1^. ATR-FTIR spectra were processed using the FITYK software (1.3.1 version) and the ratio of band intensities at 995 and 1022 cm^−1^ was determined to study the film’s molecular rearrangement.

#### 2.3.6. X-ray Diffraction (XRD) Studies

A Bruker diffractometer (D8-Advance model, Ettlingen, Germany) was used to obtain the diffractograms of the films. This equipment has a KRISTALLOFLEX K 76080F X-Ray generator (power = 3000 W, voltage = 20–60 kV, intensity = 5–80 mA), that has an X-Ray tube with a copper anode. The dimensions of specimens were 1 × 1 cm² and the equipment was operated at 40 kV and 40 mA with 2θ varying from 4 to 50° with a step size of 0.05°.

#### 2.3.7. Dynamic Mechanical Analysis (DMA)

The DMA analysis was carried out in a DMA 1 Instrument (Mettler-Toledo, Barcelona, Spain). The dimensions of the samples used for this analysis were 8.5 × 25 × 1 mm and the temperature range was −100 °C to 60 °C at a rate of 3 °C/min and a constant frequency of (1 Hz).

## 3. Results and Discussion

**Figure 1:**
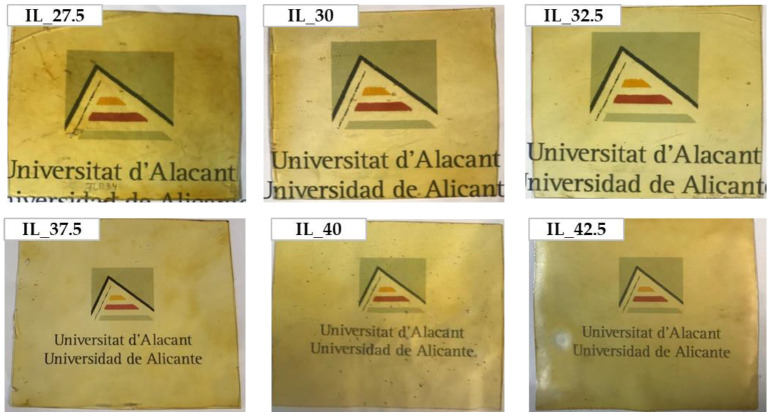
The visual aspect of starch/PVA blends plasticized by [Emim^+^][Ac^−^].

The visual appearance of the films is shown in Figure 1. As can be seen, all are homogeneous and transparent. However, the higher the [Emim^+^][Ac^−^] percentage, the higher the transparency of the samples. In addition, they show a yellowish color attributed to the color of the [Emim^+^][Ac^−^].

### 3.1. Scanning Electron Microscopy (SEM) Analysis

**Figure 2:**
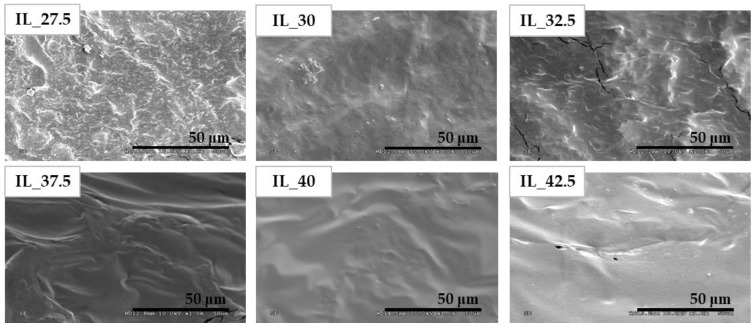
SEM images of the cryogenic fracture surface of sheets (magnification 1000×).

SEM images of the surface of the samples are shown in Figure 2. These provide useful information about the homogeneity, morphology, etc. of the films [55], features that are closely related to the final mechanical properties of the material [34].

SEM micrographs showed a heterogeneous matrix with some cracks and some readily visible starch granules that had not been dissolved during mixing and hot pressing at IL concentrations of up to 32.5%. At higher concentrations, starch is clearly destructured, dissolved, and incorporated into a continuous phase that exhibits a smooth surface in SEM images, especially in samples with 40 and 42.5% of IL.

### 3.2. Mechanical Properties

**Figure 3:**
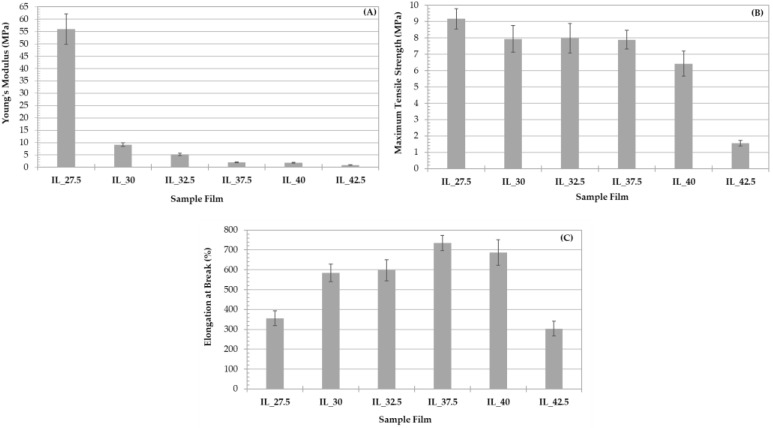
Mechanical properties of the films studied. (**A**) Young’s modulus; (**B**) Maximum Tensile Strength; and (**C**) Elongation at break.

The mechanical properties of starch/PVA with IL as a plasticizer are shown in Figure 3. From these properties, it is possible to know the potential use of the films in future applications [47,60].

According to Figure 3, the values are heavily dependent on the content of IL; the results of Young’s modulus vary from 56.1 MPa to 0.911 MPa, and the maximum tensile strength from 9.17 MPa to 1.56 MPa. In both cases, the maximum and minimum values correspond to the IL_27.5 and IL_42.5 samples, respectively. Elongation at break ranged from 735% to 304% at IL concentrations of 37.5 and 42.5%, respectively. 

Results obtained reveal a sharp drop in Young’s modulus when IL concentration is around 30%. At higher concentrations, Young’s modulus progressively decreases coinciding with a raise in elongation at break which indicates a clear plasticizing effect of the IL. Nevertheless, concentrations up to approximately 40% involve a clear excess of plasticizer, since above this value tensile strength and elongation at break markedly decrease. In fact, at these concentrations, the material starts to become a gel, extremely soft and sticky. 

In order to understand this behavior, it is important to bear in mind the effect of IL in starch. As reported in the literature, ILs are good starch solvents, but at the same time they could provoke a certain degree of depolymerization, and, more specifically, those including the ion chloride. The use of acetate does not prevent this depolymerization process since it seems to occur, even to a low extent, at concentrations of around 50% of [Emim^+^][Ac^−^] [61].

Thus, the results shown in Figure 3 could be the result of the contribution of several effects. On the one hand, as the IL content increases, starch is progressively dissolved and the blend becomes more ductile [40] as expected by the presence of the plasticizer: tensile strength drops while elongation at break increases. Above 37.5% of IL content, the complete gelatinization of the material for having an excess of plasticizer contributes to a weak interaction between the starch chains which is responsible for the drop in mechanical properties.

### 3.3. Hydration Properties

The hydration properties are the water content and the solubility in water, which are shown in Table 1. These properties are limiting and important factors of the film to determine its use in the future [62].

On the one hand, as can be observed in the table, the values obtained of moisture of films with [Emim^+^][Ac^−^] as a plasticizer show a plateau with a value close to 10% of moisture content, but drastically increases at a 40% content of IL reaching a value of moisture around 15%. On the other hand, solubility values progressively increase with IL concentration, observing solubility changes not only attributable to the IL extraction and dissolution. When IL concentration is 27.5%, around 35% of starch formulation dissolves, but when IL concentration raises 15 units up to 42.5% (i.e., 15% more), the solubility increases to 58%. In general, this behavior could be attributed in part to the hygroscopic nature of the ILs and, in particular, [Emim^+^][Ac^−^] has a high hygroscopicity and water solubility [63]. In addition, the IL could contribute to higher and stronger water–ion interactions [41,64] and, at the same time, to a certain starch depolymerization [61] which forms monosaccharides and disaccharides at high IL contents [65]. This phenomenon is due to the alkyl chain length of cations, meaning the hydrophobicity of the ILs increases as the increase in the alkyl chain length of the cations [2,5].

It is worth mentioning that water solubility is related to the biodegradability of the material; the material’s biodegradability should increase with the solubility [58]. However, there are applications where the material has to be insoluble to preserve the integrity of the final product [66].

### 3.4. Migration Degree

The migration degree of plasticizers is an interesting parameter to bear in mind because it can determine the life of the material since this phenomenon is an undesirable effect that causes difficulties in its commercialization and uses in long-term applications [59]. The migration is due to starch retrogradation or recrystallization [67], which involves changes from the amorphous state of starch to new ordered structures [68]. In addition, the migration process depends on nature, molecular weight, and amount of plasticizer, and this in turn results in the loss of material properties [59] because when it is produced for plasticizer loss in TPS, this causes a decrease of elongation at break and an increase in the elastic modulus [69,70].

**Figure 4:**
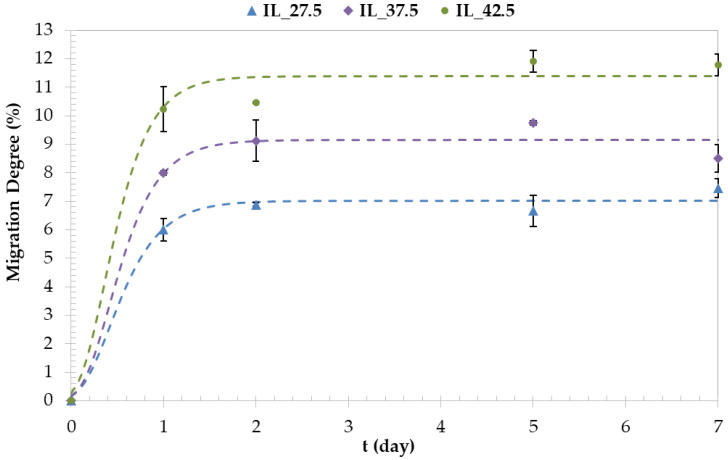
Migration degree as a function of time and [Emim^+^][Ac^−^] content of starch/PVA blends.

The values of IL migration are shown in Figure 4. These results follow a clear trend since the migration of IL increases as the plasticizer amount increases. Hence, a small content of [Emim^+^][Ac^−^] as a plasticizer in the starch/PVA blends helps to reduce this effect (IL_42.5 and IL_27.5 have 11.8% and 7.46% of migration degree of plasticizer, respectively). It must be taken into account that the superficial moisture of the samples has probably been eliminated during this test. However, this fact does not change the results or the trend obtained from the migration test. Analyzing these, the plasticizer migration mainly occurs in the early 24 h of the test.

As far as we know, there are no scientific findings in the literature on the migration of starch/PVA films plasticized by any IL. Nevertheless, Rahman et al. [9] studied the migration in samples of PVC plasticized by different ILs and concluded that the PVC with traditional plasticizers had a greater weight loss than those that use ILs as a plasticizer, which is due to the bulky structure of some ILs that hinders their migration to the surface of the material. They also carried out the study of plasticizer loss due to leaching. These can be considered similar methods and their obtained results are comparable because the aim in both cases is to quantify the plasticizer loss; the water is used as an extractant in the leaching experiment, while migration samples are subjected to a determinate pressure. The same trend was observed in the percentage of plasticizer loss to that shown in Figure 4 and they confirmed that using IL as a plasticizer reduced the leaching.

Therefore, we could conclude that the problem of plasticizer migration to the surface of the films is reduced when using a small percentage of certain ILs as plasticizers in starch/PVA blends.

### 3.5. Attenuated Total Reflection-Fourier Transform Infrared (ATR-FTIR) Spectroscopy

**Figure 5:**
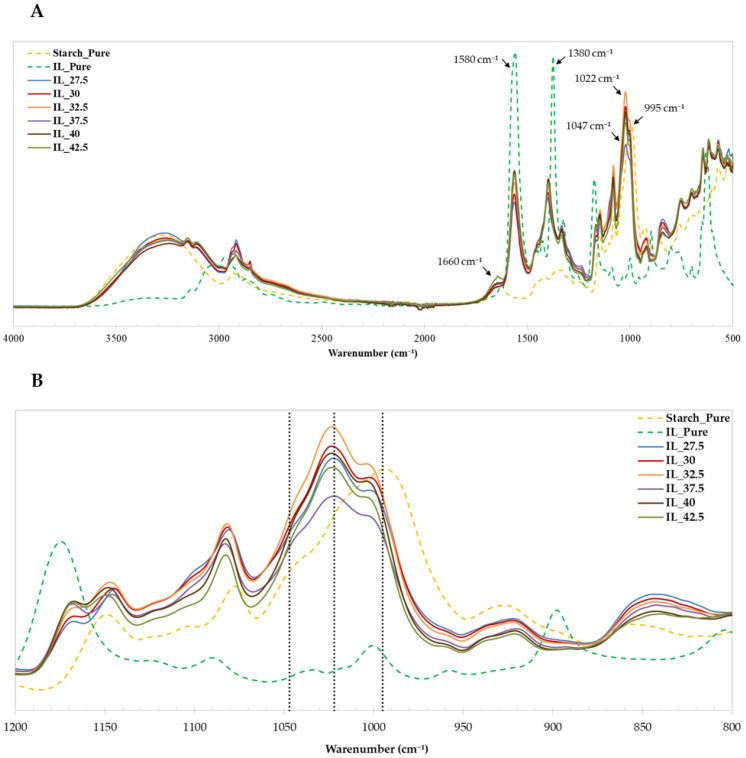
ATR-FTIR spectra of starch/PVA blends plasticized by [Emim^+^][Ac^−^]. (**A**) 500–4000 cm^−1^ range; (**B**) 800–1200 cm^−1^ range.

Figure 5 shows the ATR-FTIR spectra of the samples. This technique reflects the changes in molecular interactions in the starch/PVA films with [Emim^+^][Ac^−^] as a plasticizer. As can be seen, the spectra are similar and, as expected, show the corresponding peaks to IL and starch.

First of all, it is worth mentioning a wide band between 3000–3600 cm^−1^ where two different bands overlap: on the one hand, the stretching vibration of the –OH groups of the glucose units and water [60] and the vibration C–H of the imidazolium ring in [Emim^+^][Ac^−^] [71].

Other bands shared by both components (starch and [Emim^+^][Ac^-^]) are localized between 2800–3000 cm^−1^, and this is attributed to asymmetric and symmetric stretching of –CH_2_ and –CH_3_, and aliphatic asymmetric C–H stretching vibrations [47].

The infrared spectrum of the pure IL reveals two peaks at 1380 and 1580 cm^−1^ corresponding to symmetric and asymmetric O–C–O stretches of [Ac^−^] anion of the IL, respectively [72]. However, in Figure 5, those peaks are shown at 1400 and 1560 cm^−1^, slightly shifted to the position in the pure IL, probably due to the result of the hydrogen bonding between the IL and water molecules (which presents in the starch as moisture) [73]. In addition, the small peak around 1660 cm^−1^ is assigned to water [47] or the –C–H stretching of the vinyl group [74].

The band at 1148–925 cm^−1^ shows the stretching of –C–O– situated in –C–O–H and –C–O–C– bonds of the glucose of starch [47]. More concretely, the peaks at around 995 cm^−1^, 1022 cm^−1^, and 1047 cm^−1^ are related to the degree of the rearrangements of the starch after the process of plasticization. While the band at 995 cm^−1^ corresponds to the hydrogen bonds and the regularity given by molecular rearrangements, the peak at 1022 cm^−1^ is associated with the amorphous phase of the films, and a decrease in crystallinity [26,33,50,74]; the degree of order in starch can be quantified in consequence by the ratio of the areas of the bands at 995 and 1022 cm^−1^ [47].

The results are shown in Table 2. According to the ratio values calculated, it can be seen that R_995/1022_ decreases when increasing the IL content (IL_27.5 and IL_42.5 have values of 0.824 and 0.536, respectively), indicating the ability of this IL to hinder molecular rearrangements after plasticization. These results are in good agreement with the XRD and DMA, as shown below.

### 3.6. X-ray Diffraction (XRD) Studies

**Figure 6:**
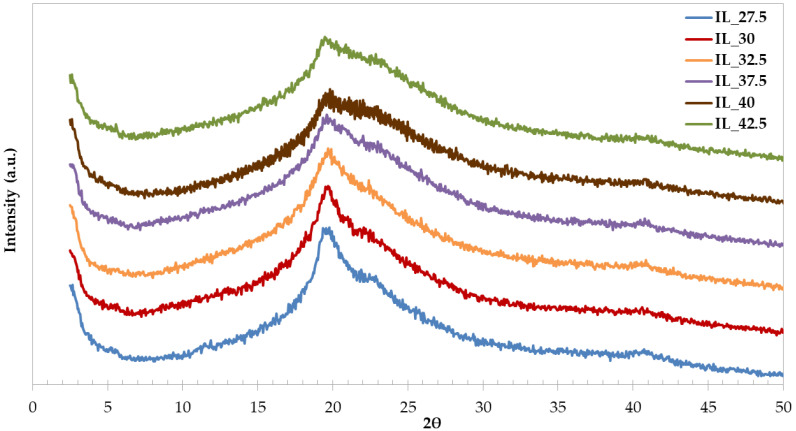
XRD spectra of the starch/PVA blends plasticized by [Emim^+^][Ac^−^].

The XRD spectra of the starch/PVA blends plasticized by [Emim^+^][Ac^−^] are shown in Figure 6. This analysis allows the study of the crystalline structure of films. All samples showed a similar pattern, where it is possible to observe peaks at around 20, 23, and 40° with different intensities; the peak at 2Ɵ = 20° is less prominent as [Emim^+^][Ac^−^] content increases, so a loss of crystallinity is observed. This fact is due to the plasticization process since when the IL content increases the disruption of hydrogen bonds between the starch molecules [75,76], this increases the mobility of the starch chains and contributes to the creation of a more amorphous structure, according to the conclusions of Domene-López et al. [47,55] and Luchese et al. [32], who also studied the native starch and the starch films using [Emim^+^][Ac^−^] as a plasticizer, obtaining similar results. Xie et al. [40] concluded that using IL as a plasticizer contributed to a decrease in crystallinity, in comparison with glycerol, because the [Emim^+^][Ac^−^] decreases the B-type crystallinity and the V-type crystallinity, hindering the formation of the single-helical structure due to interaction between the hydroxyl groups of starch and acetate anion in [Emim^+^][Ac^−^].

It is worth mentioning that various authors such as Zhang et al. [26], Bhagabati et al. [77], and Domene-López et al. [47] state that the transparency of the final material is related to its crystallinity; more amorphous polymeric films had higher transparency due to the disruption of starch grains, and this effect was observed in the obtained films (see Figure 1); when increasing the IL content, the final films lose their crystalline structure, but they are more transparent. 

### 3.7. Dynamic Mechanical Analysis (DMA)

**Figure 7:**
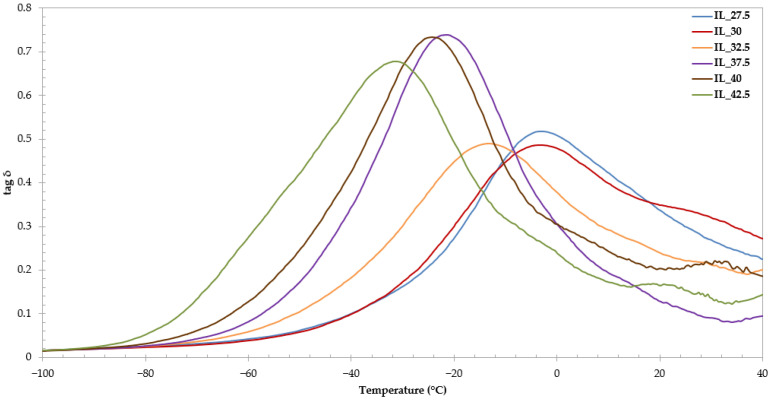
DMA spectra of the studied sheets. Dependence of loss tangent (tan δ) on the temperature at a constant frequency of 1 Hz.

The DMA data of the starch/PVA[Emim^+^][Ac^−^] are shown in Figure 7. The curves are similar, showing a peak corresponding to the glass transition of the system [15,78]. As can be seen, the glass transition of samples is shifted towards lower temperatures when IL content increases; for example, the T_g_ of IL_27.5 was −4 °C while the T_g_ of IL_42.5 was −30 °C. Jaramillo et al. [58] concluded that this was associated with the plasticization process. In addition, the peak is more prominent as the IL content increases, in good agreement with Xie et al. [40]. Thus, a higher content of IL resulted in a more amorphous structure, hence with greater mobility. This loss of crystallinity is also reflected in ATR-FTIR and XRD spectra, as commented above. These data are consistent with the mechanical properties since a decrease in the glass transition temperature with IL content produces less stiff samples with lower Young’s modulus (Figure 3A).

It should be kept in mind that moisture content (favored by the presence of IL) could also affect glass transition, as Madrigal et al. [78] reported, showing a relation between the moisture contents and the value of tan δ; they concluded that the curves showed a more prominent peak as the water content increased. As is possible to observe in Table 1, the highest results of moisture correspond to the samples with more prominent peaks (IL_37.5, IL_40, and IL_42.5).

It is worth mentioning that there is not much literature on the DMA of starch films plasticized by ILs. However, the DMA analysis of the TPS plasticized by glycerol among other plasticizers has been extensively studied in the literature [79,80,81,82] and all the curves of DMA showed two peaks (one attributed to a phase rich in plasticizer and the other in starch) while that in the [Emim^+^][Ac^−^] plasticized-starch films appear in only one transition. As mentioned above, water and conventional starch plasticizers do not bring about a full starch dissolution and gelation; it could be the reason for the presence of two different glass transitions readily observable in glycerol-starch compounds. In the case of the IL-starch, they seem to be fully dissolved and it could be the reason for the presence of one single glass transition. 

## 4. Conclusions

In the present work and, for the first time, starch/PVA films have been plasticized by a single plasticizer being an ionic liquid by melt-mixing.

According to the results obtained, it is possible to conclude that the 1-ethyl-3-methylimidazolium acetate ([Emim^+^][Ac^−^]) is an efficient plasticizer to obtain these biomaterials. After the analysis of the results, it has been possible to prove that this IL provokes the disruption of hydrogen bonds between the starch molecules, changing its crystalline structure to an amorphous state, which is possible to observe in the XRD spectra. This was also confirmed by the SEM images, which show that the presence of IL involves the dissolution of the starch granules, even when the [Emim^+^][Ac^−^] content in the films was low. Furthermore, a clear trend has been found in accordance with the IL amount added to the blends that the mechanical properties were significantly changed; when there are more crystalline domains than amorphous, the samples were stiffer (increasing Young’s modulus and maximum tensile strength). By contrast, the samples showed more flexibility when the amorphous phase was predominant, due to the increase in chain movement. [Emim^+^][Ac^−^] is a good starch plasticizer as revealed by the drop in T_g_ when the IL content increases; the IL_27.5 sample has a glass transition temperature at −3.17 °C, while the IL_42.5 sample has −31.4 °C, finding a difference of 28.2 °C between both. The same occurs in the diffusion and hydration properties, which are higher as the IL content increases; the values of IL_27.5 and IL_42.5 for the migration degree of plasticizer and solubility in water are 7.46–11.8% and 35.2–57.6%, respectively.

For all these reasons, the [Emim^+^][Ac^−^] could be considered an interesting plasticizer to obtain biodegradable polymeric blends from starch, constituting an alternative to other conventional plasticizers such as water or polyols. The results achieved through a technique prior to industrial development, such as melt-mixing, show the high potential of these materials to be a real replacement to conventional polymer in a wide range of applications such as packaging and provide the possibility to be used in applications which previously had no place in this type of biomaterial, such as in dye-sensitized solar cells, fuel cells, among others.

## Figures and Tables

**Table 1:** Hydration properties of the sheets studied.

Sample	Water Content (%)	Solubility in Water (%)
IL_27.5	9.41 ± 0.25	35.2 ± 0.2
IL_30	9.30 ± 0.26	37.0 ± 0.4
IL_32.5	9.93 ± 0.29	40.8 ± 0.2
IL_37.5	9.97 ± 0.16	49.3 ± 1.2
IL_40	11.4 ± 0.2	50.8 ± 1.0
IL_42.5	14.9 ± 0.9	57.6 ± 1.5

**Table 2:** ATR-FTIR band intensity ratio (R_995/1022_) of the starch/PVA blends obtained.

	Films Sample						
	Starch_Pure	IL_27.5	IL_30	IL_32.5	IL_37.5	IL_40	IL_42.5
R_995/1022_	1.39	0.824	0.673	0.659	0.637	0.613	0.536

## Data Availability

Not applicable.
